# SARS-CoV-2 Altered Hemorheological and Hematological Parameters during One-Month Observation Period in Critically Ill COVID-19 Patients

**DOI:** 10.3390/ijms232315332

**Published:** 2022-12-05

**Authors:** Daniel Alexander Bizjak, Lucas John, Lynn Matits, Alisa Uhl, Sebastian Victor Waldemar Schulz, Jana Schellenberg, Johannes Peifer, Wilhelm Bloch, Manfred Weiß, Beate Grüner, Hendrik Bracht, Jürgen Michael Steinacker, Marijke Grau

**Affiliations:** 1 Division of Sports and Rehabilitation Medicine, University Hospital Ulm, 89075 Ulm, Germany; 2 Clinical & Biological Psychology, Institute of Psychology and Education, Ulm University, 89081 Ulm, Germany; 3 Central Emergency Services, University Hospital Ulm, 89081 Ulm, Germany; 4 Institute of Cardiovascular Research and Sports Medicine, Molecular and Cellular Sports Medicine, German Sport University Cologne, 50933 Cologne, Germany; 5 Clinic for Anaesthesiology and Intensive Care Medicine, University Hospital Medical School, 89081 Ulm, Germany; 6 Department of Internal Medicine III, Division of Infectious Diseases, University Hospital Ulm, 89081 Ulm, Germany

**Keywords:** COVID-19, hemorheology, RBC deformability, aggregation, inflammation, thrombosis, hematology, SARS-CoV-2, erythrocyte alterations

## Abstract

Hematological and hemorheological parameters are known to be altered in COVID-19; however, the value of combined monitoring in order to deduce disease severity is only scarcely examined. A total of 44 acute SARS-CoV-2-infected patients (aCOV) and 44 age-matched healthy controls (Con) were included. Blood of aCOV was sampled at admission (T0), and at day 2 (T2), day 5 (T5), day 10 (T10), and day 30 (T30) while blood of Con was only sampled once. Inter- and intra-group differences were calculated for hematological and hemorheological parameters. Except for mean cellular volume and mean cellular hemoglobin, all blood cell parameters were significantly different between aCOV and Con. During the acute disease state (T0–T5), hematological and hemorheological parameters were highly altered in aCOV; in particular, anemic conditions and increased immune cell response/inflammation, oxidative/nitrosative stress, decreased deformability, as well as increased aggregation, were observed. During treatment and convalescence until T30, almost all abnormal values of aCOV improved towards Con values. During the acute state of the COVID-19 disease, the hematological, as well as the hemorheological system, show fast and potentially pathological changes that might contribute to the progression of the disease, but changes appear to be largely reversible after four weeks. Measuring RBC deformability and aggregation, as well as oxidative stress induction, may be helpful in monitoring critically ill COVID-19 patients.

## 1. Introduction

It has been almost three years since the coronavirus disease 2019 (COVID-19), induced by the severe acute respiratory syndrome coronavirus 2 (SARS-CoV-2), was first described. Still, and possibly caused by diverse mutations, infected individuals exhibit new and highly diverse symptoms. After infection, some individuals may remain asymptomatic or suffer from mild upper respiratory symptoms, whereas others develop life-threatening symptoms such as severe acute respiratory distress syndrome (ARDS), pneumonia, cardiac, vascular, and organ damage, or neurological diseases [1]. A recently published model based on literature studies describes four stages during the first 10–15 days after infection [2]. The first stage of COVID-19 is characterized by upper respiratory tract infection and symptoms such as cough, sore throat, fever, and fatigue. In the second stage, patients suffer from dyspnoea and pneumonia. The third stage marks the exacerbation of the clinical scenario with severe complications dominated by a cytokine storm and local and systemic hyperinflammatory state. Next to serious lung lesions and ARDS, caused by vasculopathy and thrombosis, acute cardiac and renal damage, as well as sepsis and secondary infections, are most frequently reported in this stage. Finally, the fourth stage is characterized by either death or recovery [2]. Mortality is associated with, e.g., advanced age and comorbidities such as hypertension, diabetes, and cardiovascular or chronic lung diseases [2].

Especially in the acute phase of the disease, the hematological and inflammatory profile is altered in COVID-19. Among others, increased white blood cell count (particularly neutrophil count), erythrocyte sedimentation rate (ESR), C-reactive protein (CRP), D-dimer, and fibrinogen concentrations were observed and correlated with disease severity and mortality [3,4]. Increased fibrinogen and D-Dimer concentrations are frequently observed as by-products of inflammation and gut perforation in COVID-19 with subsequent negative effects on the coagulation system [5,6]. Blood clot dissolving by plasmin leads to degradation products, including D-dimers, which in high concentrations may interact with red blood cells (RBCs), resulting in fragile RBC membranes and pathological RBC elasticity [7]. This can result in RBCs trapping in embolisms and clots formed in COVID-19 patients, as decreased platelet and lymphocyte concentrations were found to increase the risk of a severe COVID-19 phenotype and a high risk of anemia [4,8].

In line with these hematological observations, many patients show cold extremities and weak peripheral pulses, which might indicate microcirculatory dysfunction [9]. In addition, particularly the shortness of breath and the reduced performance capability might be caused by altered oxygen uptake in RBCs, oxygen binding, and/or oxygen release by the RBCs [10,11].

RBC deformability is an important parameter that is related to RBC longevity and that has been described to affect blood flow and the efficacy of oxygen transport [12]. It was demonstrated that RBCs express an isoform of the nitric oxide synthases (RBC-NOS) that can improve RBC deformability by producing NO that can bind to the cytoskeleton of the RBC membrane [13]. The activation of the RBC-NOS can be modulated by versatile mechanisms, including oxidative stress, RBC aging, and health status [14,15]. RBC aggregation is another essential parameter that affects blood viscosity and microvascular flow dynamics [16], which is also affected by different forms of hereditary or virus-driven diseases [17,18,19], but up to now has not been examined during the COVID-19 disease development over time.

Recent investigations demonstrated impaired rheological properties of COVID-19 RBCs compared to healthy control RBCs [12,19], including lower deformability, increased blood viscosity, lower hematocrit (Hct) as well as increased RBC aggregation, which were associated with clinical complications, and which could consequently contribute to clinical manifestations such as reduced performance capacity because of impaired oxygen transport and delivery.

Previous investigations focused on the acute changes related to COVID-19, while the present study aims to monitor the hematological and hemorheological parameters of COVID-19 patients during the first thirty days of intensive care treatment. This investigation may contribute to understanding the disease progression and, thus, might be helpful in diagnosis and severity assessment during the acute infection and rehabilitation phase.

## 2. Results

### 2.1. Study Population and Blood Samples

**Figure 1:**
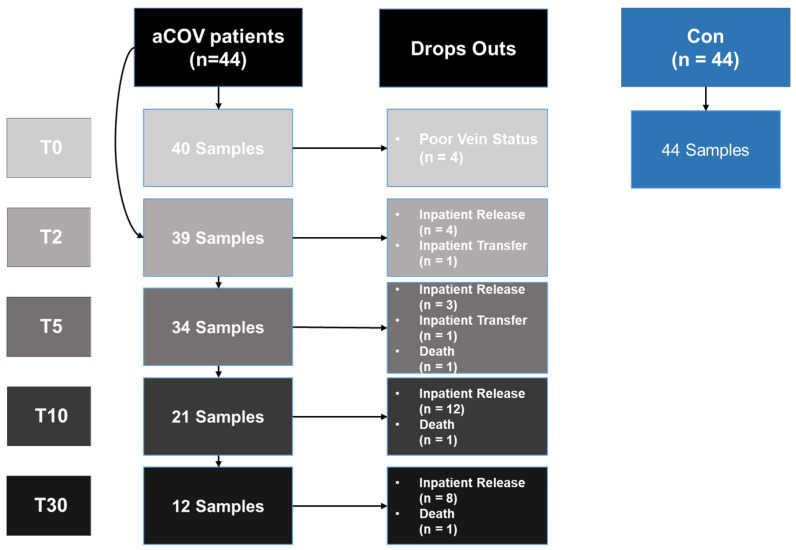
Blood sample collection from the COVID-19 patients (aCov) and the healthy control group (Con) at the respective time point (i) acute/admission (T0), (ii) day two (T2), (iii) five (T5), (iv) ten (T10) and (v) thirty (T30) after admission. Sample number decreased during the study due to poor vein status, inpatient release/transfer, or death (Drop-Outs). In detail, some patients were lost due to patient inaccessibility after inpatient release/transfer (non-cooperating hospital/physician; inability to travel to the hospital again after patient release at the respective definite time points). The majority of the enrolled patients visited the Division of Sports and Rehabilitation Medicine for further blood sampling time points after hospital admission. Some of the 44 included samples were collected for the first time at T2, explaining the number discrepancy between T0 and T2. Con samples were collected once only.

General characteristics, including vaccination status and inpatient stay time, are presented in Table 1. During the course of the study, three patients died, one before the blood sample collection on T5, one before T10, and one before T30. Moreover, blood sampling was also not possible at some time points because of inpatient release, blood volume restrictions in the severely clinically ill as well as retrospectively included patients after T2. In total, 40 samples were measured at T0, 39 samples at T2, 34 samples at T5, 21 samples at T10, and 12 samples at T30. Overall, complete data sets are available for nine patients (Figure 1).

### 2.2. Hematological Parameters

#### 2.2.1. Red Blood Cell Variables

**Figure 2:**
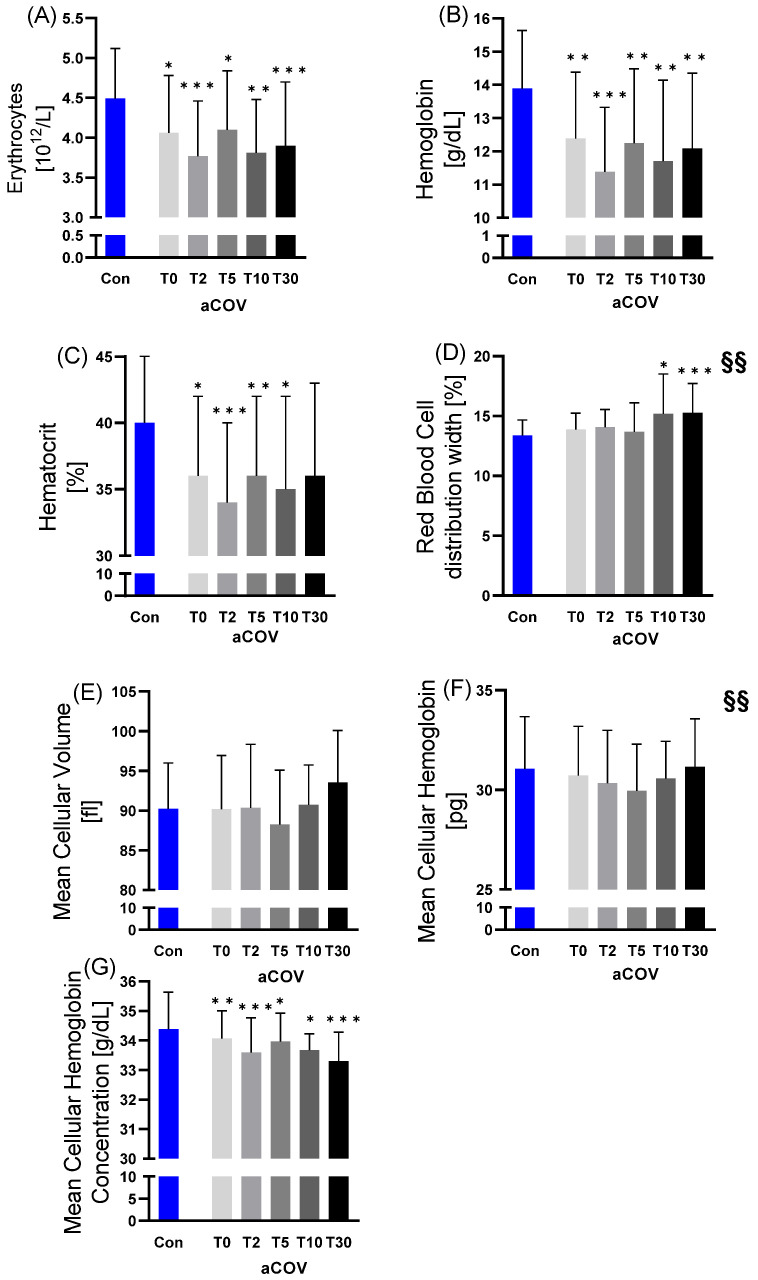
Red blood cell (RBC) variables assessed for acute COVID-19 infected individuals (aCOV) at different time points at the intensive care unit (T0 hospital admission; T2 day 2; T5 day 5; T10 day 10; T30 day) compared to healthy controls (Con). While (**A**) RBC concentration, (**B**) Hemoglobin (Hb) concentration, (**C**) Hematocrit (Hct), and (**G**) MCHC were decreased in aCOV compared to Con, (**D**) RDW (at T10 and T30) showed increased values. (**E**) MCV and (**F**) MCH of aCOV were not different compared to Con. A change of values over time was observed for RDW (increase) and MCH (U-shaped). * *p* ≤ 0.050; ** *p* ≤ 0.010; *** *p* ≤ 0.001 compared to Con; §§ *p* ≤ 0.010 changes over time in aCOV.

RBC, mean cellular hemoglobin concentration (MCHC), and hemoglobin (Hb) concentrations were lower at all time points in aCOV compared to Con. Additionally, Hct at T0, T2, T5, and T10 were lower in aCOV than in Con. In contrast, mean cellular volume (MCV) and mean cellular hemoglobin (MCH) showed no difference between the groups. Red blood cell distribution width (RDW) was significantly increased in aCOV at T10 (15.2%; *p* < 0.050) and T30 (15.3%; *p* < 0.001) compared to Con (13.4%). (Figure 2).

In aCOV, MCH (J-shaped; *p* < 0.010) and RDW (increasing; *p* < 0.010) showed significant changes during the 30 days of treatment.

#### 2.2.2. Immune Cell and Inflammatory Variables

**Figure 3:**
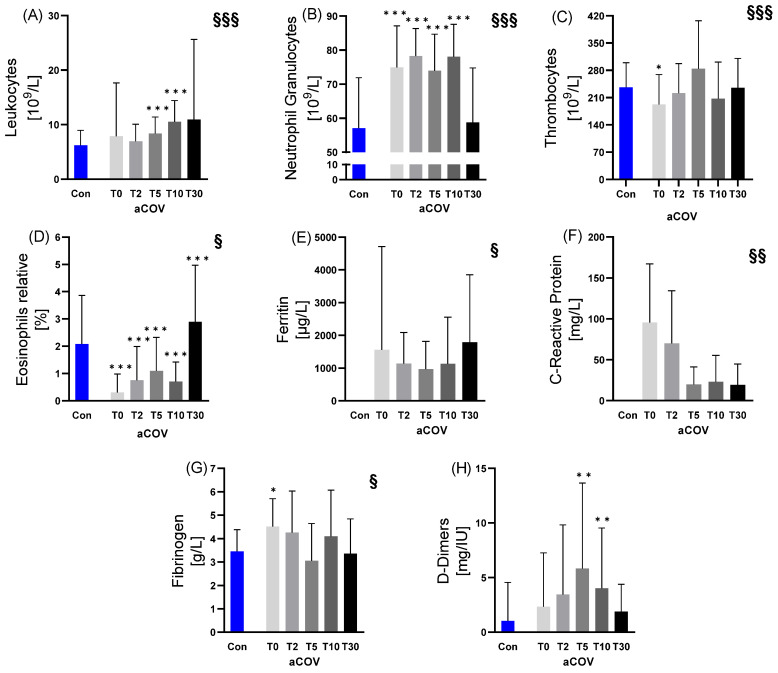
Immune cell and inflammation variables measured in acute COVID-19 infected individuals (aCOV) at different time points at the intensive care unit (T0 hospital admission; T2 day 2; T5 day 5; T10 day 10; T30 day) compared to healthy controls (Con). While (**A**) Leukocytes at T5 and T10, as well as (**B**) Neutrophil Granulocytes at T0–T10 were increased in aCOV compared to Con, (**C**) Thrombocytes were decreased at T0. The (**D**) relative proportion of Eosinophils was decreased between T0–T10 and increased at T30. (**G**) Fibrinogen at T0 and (**H**) D-Dimers (at T5 and T10) were increased compared to Con, and fibrinogen showed a significant change over time. Due to blood volume restrictions, no measurements could be made for (**E**) Ferritin and (**F**) CRP in Con, but intragroup analysis of aCOV showed for both, Ferritin and CRP, a significant change over time as well as highly increased values compared to clinical reference values [20,21]. * *p* ≤ 0.05; ** *p* ≤ 0.01; *** *p* ≤ 0.001 compared to Con; § *p* ≤ 0.05; §§ *p* ≤ 0.01; §§§ *p* ≤ 0.001 changes over time in aCOV.

Compared to Con, neutrophil granulocytes (T0, T2, T5, T10), leukocytes (T5, T10), thrombocytes (T0), relative eosinophils (all time points), and fibrinogen concentration (T0) were significantly different in aCOV. D-dimers were higher at T5 and T10, respectively (Figure 3).

Regarding the changes in immune cells, inflammatory and coagulation variables over the study period of 30 days, leukocytes (increase; *p* < 0.001), neutrophil granulocytes (bell-shaped T0–T5, increase T5–T10, decrease T10–T30; *p* < 0.001), thrombocytes (bell-shaped T0–T10, increase T10–T30; *p* < 0.001), relative eosinophils (bell-shaped T0–T10, increase T10–T30; *p* < 0.050), ferritin (U-shaped; *p* < 0.050), CRP (decrease; *p* < 0.010), and fibrinogen (decrease T0–T5, increase T5–T10, decrease T10–T30; *p* < 0.050) showed significant effects over the study period of 30 days. In aCOV, ferritin values (1789 µg/L) and peak CRP values (95.5 mg/L) were approximately five-fold [20] and nine-fold higher [21] compared to clinical reference values. 

### 2.3. Hemorheological Parameters and RBC Immunostaining

#### 2.3.1. RBC Deformability, RBC-NOS Activation state and Nitrotyrosine Levels

**Figure 4:**
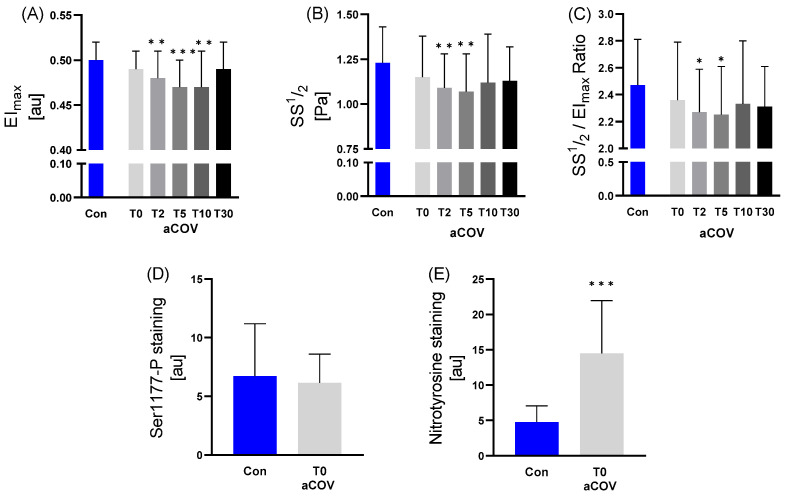
Deformability variables assessed for acute COVID-19 infected individuals (aCOV) at different time points at the intensive care unit (T0 hospital admission; T2 day 2; T5 day 5; T10 day 10; T30 day) compared to healthy controls (Con). In aCOV, values for (**A**) EI_max_ (T2, T5 and T10), (**B**) SS½ (T5 and T10) and (**C**) SS½/EI_max_ ratio (T5 and T10) were significantly lower compared to Con. (**D**) RBC-NOS activation indicated by serine1177 phosphorylation was not different in aCOV compared to Con, whereas (**E**) RBC nitrotyrosine staining in aCOV showed higher values. There was no significant change observed during the 30 days of treatment in the deformability variables. Due to the limited staining numbers, a comparison between Con (n = 29) and aCOV (n = 9) was only performed for T0. * *p* ≤ 0.05; ** *p* ≤ 0.01; *** *p* ≤ 0.001 compared to Con.

Compared to Con, the maximal Elongation Index EI_max_ of aCOV was decreased at T2 (*p* = 0.006), T5 (*p* < 0.001), and T10 (*p* = 0.002), respectively, while the shear stress at half-maximal deformability SS½ was different at T2 (1.09 vs. 1.23; *p* < 0.010) and T5 (1.07 vs. 1.23; *p* < 0.010). These differences were also observed for SS½/EI_max_ (2.27 vs. 2.47, T2 *p* < 0.050; 2.25 vs. 2.47, T5 *p* < 0.050) (Figure 4).

Due to blood volume restrictions and sample quality, comparisons for RBC staining were performed only for basal values of Con (n = 29) and aCOV’s hospital admission time point T0 (n = 9).

While there was no difference for RBC-NOS Serine1177 phosphorylation (activation state of the enzyme) between Con and aCOV (*p* = 0.658), nitrotyrosine staining was significantly higher in aCOV compared to Con (*p* < 0.001) (Figure 4).

#### 2.3.2. RBC Aggregation

**Figure 5:**
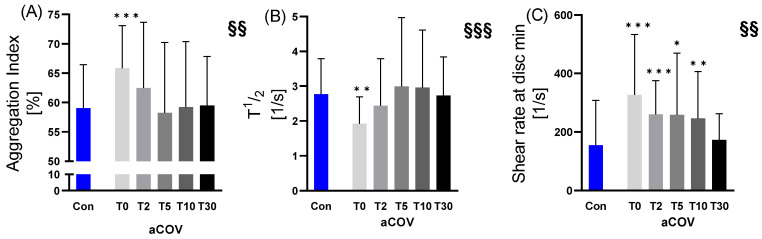
Aggregation parameters assessed for acute COVID-19 infected individuals (aCOV) at different time points at the intensive care unit (T0 hospital admission; T2 day 2; T5 day 5; T10 day 10; T30 day) compared to healthy controls (Con). While for (**A**) the Aggregation Index AI increased values were observed at hospital admission T0 in aCOV compared to Con, (**B**) decreased values in T^1^/_2_ were seen at the same time point. (**C**) The shear stress at disc min—minimum change in back-scatter intensity found during the iteration procedure—was increased between time points T0–T10 compared to Con. A change of values over time was observed for AI, and T^1^/_2,_ the shear stress at disc min. * *p* ≤ 0.05; ** *p* ≤ 0.01; *** *p* ≤ 0.001 compared to Con; §§ *p* ≤ 0.01; §§§ *p* ≤ 0.001 changes over time in aCOV.

Compared to Con (AI 59.01%; T½ 2.77 ^1^/_s_), the aggregation Index AI (65.83%; *p* = 0.001), as well as T½ (1.92 ^1^/_s_; *p* = 0.004) of aCOV, were different at T0, while the shear rate at dIsc min differed at all time points (T0 *p* < 0.001, T2 *p* < 0.001, T5 *p* < 0.050, T10 *p* < 0.010), except for T30 (*p* = 0.202) (Figure 5).

Regarding changes of aCOV aggregation variables over time, AI (J-shaped; *p* < 0.050), T½ (bell-shaped; *p* < 0.010), and shear rate at dIsc min (decrease; *p* < 0.010)) showed significant changes over time.

The detailed statistical analysis of all hematological and hemorheological variables with all respective *p*-values can be found in Supplementary Materials File S1 (Supplementary Tables S1–S3).

## 3. Materials and Methods

### 3.1. Study Population and Study Design

The observational, non-interventional CEDRIC (Changes of Erythrocytes During COVID-19 Infection) study was carried out between August and December 2021 at the University Hospital Ulm and was a cooperation study involving the Division of Sports and Rehabilitation Medicine, the Department of Infectiology, the Central Emergency Department, the Intensive Care Unit (ICU), the Clinical Immunology, and the German Sport University Cologne. The study was approved by the ethics committee of Ulm University (AZ 255–21), registered at the German register for clinical trials (trial number DRKS00026707), and was performed in accordance with the Declaration of Helsinki. All participants gave written informed consent to participate in this study.

On admission to the ICU, patients with COVID-19 who needed intensive care treatment were recruited. Inclusion criteria included an age ≥ 18 years, a proven acute SARS-CoV-2 infection, and typical clinical symptoms related to the disease (e.g., sore throat, shortness of breath, headache, etc.). All subjects who stated that they refused blood sampling or did not provide written informed consent were excluded. In addition, patients with acute comorbidities (e.g., severe blood loss, severe iron deficiency, dialysis) that could influence the blood variables analyzed were excluded.

In total, 44 acutely infected COVID-19 patients (aCOV; 19 females, 25 males; 59.0 ± 16.9 years of age) fulfilled the inclusion criteria and were included in the study. A standard COVID blood sampling (7.5 mL Ethylenediaminetetraacetate (EDTA) anticoagulated blood (S-MONOVETTE^®^, Sarstedt, Nümbrecht, Germany)) was taken from the arm vein after confirmed consent, which was supplemented by the blood samples necessary for the laboratory test (7.5 mL serum and 2.7 mL citrate (S-MONOVETTE^®^, Sarstedt, Nümbrecht, Germany)).

Blood samples were taken at five time points: (i) patient admission at ICU defined as Acute Time point (T0), (ii) days two (T2), (iii) five (T5), (iv) ten (T10), and (v) thirty (T30) after admission, respectively. 

The control group consisted of 44 age-matched healthy individuals (Con; 24 females, 20 males; 52.1 ± 18.1 years of age) without acute infection at the time of blood sampling and without comorbidities that could affect the blood cell parameters.

Blood samples were taken once from the control group: 7.5 mL EDTA blood, 7.5 mL serum blood, and 2.7 mL citrate blood (S-MONOVETTE^®^, Sarstedt, Nümbrecht, Germany). Blood samples were processed within 6 h after sampling. The blood samples of the study group and the control group were examined in exactly the same way.

### 3.2. Hematological Parameters

The following parameters were measured using EDTA anti-coagulated whole blood:

Neutrophil granulocytes (*10^9^/L), RBCs (*10^12^/L), thrombocytes (*10^9^/L), relative numbers of eosinophils (%), leukocytes (*10^9^/L), RDW (%), Hct (%), Hb concentration (g/dL), MCH (pg), MCHC (g/dL), MCV (fl), C-reactive protein (CRP) (mg/L), ferritin (µg/L), D-dimers (mg/IU), and fibrinogen concentration (g/L). Parameters were determined and analyzed by clinical standards using the Sysmex XE-5000 analyzer (Sysmex, Hamburg, Germany) system (based on resistance measurement principle (impedance measurement, Coulter measurement principle), photometric measurement, differentiation in a flow cell by means of laser via VCSn technology (volume, conductivity, scatter)) as well as ECLIA (Roche Immunoassay Analyzer Cobas 8000, Cobas t 711 and Cobas t 511, Basel, Switzerland) and ELISA measurements.

### 3.3. Deformability Measurement

RBC deformability was measured using the Laser-Assisted Optical Rotational Cell Analyzer (LORRCA, RR Mechatronics, Hoorn, The Netherlands). Briefly, 1 mL of RBC suspension (10 μL of whole blood in 2.5 mL of isotonic 0.14 mM polyvinylpyrrolidone (PVP) solution (osmolarity 300 mOsM/L, viscosity 28.7 mPa*sec at 37 °C)) was placed between two concentric cylinders at physiological pH 7.4 and 37 °C. The rotation of the outer cylinder around an inner static cylinder yields, in specific, shear forces acting on the RBCs, which forces the deformation of the cells. A laser beam that passes through the cell suspension was diffracted by the RBCs, and the resulting diffraction pattern was analyzed by the LORRCA software [22,23]. Nine consecutive shear stresses between 0.3 and 30 Pa (0.30, 0.53, 0.95, 1.69, 3.00, 5.33, 9.49, 16.87, 30.00) were applied per measurement, and width (W) and length (L) of the diffraction pattern being detected. The results were expressed as an Elongation Index (EI): EI = (L − W)/(L + W). EI values were plotted as a Michaelis--Menten-like function, and the maximal Elongation Index (EI_max_) was determined. EI_max_ describes the theoretical maximum deformability at infinite shear stress. In addition, SS½, representing shear stress required for half-maximal deformability, and SS½ to EI_max_ ratio were calculated. High SS½ and SS½ to EI_max_ ratio indicate reduced deformability [24,25,26,27].

### 3.4. Aggregation Measurement

Native samples were oxygenated for at least 15 min. The LORRCA was used to measure RBC aggregation by syllectometry. A syllectogram was created by plotting the laser backscattered intensity (Isc), measured by a photodiode, versus time. By plotting the Isc on a logarithmic scale, the LORRCA software allows to determine the Aggregation index (AI), defined as: AI = [Surface A/Surface (A + B)] [28].

In addition, T^1^/_2_ (sec) (Time that elapses until the peak intensity is reduced by half the amplitude) and Shear Rate at disc min (^1^/_s_) (minimum change in back-scatter intensity found during the iteration procedure) were obtained from the syllectrogram.

In syllectometry, blood was illuminated and subjected to shear stress, causing the cells to deform and align in the direction of the flow, while the change in backscattered light was measured after the driving mechanism stopped. In the resulting time-dependent intensity plot, the so-called syllectogram, four RBC-behavioral stages were distinguished: (1) The initial plateau during the disaggregation stage originates from the light that is backscattered by the elongated RBCs that are aligned in the direction of the flow. (2) The shape-recovery stage that follows immediately after cup cessation. (3) Aggregation state that starts during the shape-recovery stage when external shear forces fail to keep the RBCs dissociated (rouleaux formation). (4) So-called 3D aggregate formation following rouleaux formation during which rouleaux connect end-to-end as well as side-to-end, creating larger 3D aggregates [29].

### 3.5. Immunostaining Protocol for the Detection of RBC-NOS Activation State and Nitrotyrosine

Published immunostaining protocols were used to investigate RBC-NOS activation by analysis of the serine 1177 phosphorylation and to investigate nitrotyrosine staining. First, RBCs from whole blood were immediately treated with 4% formaldehyde in order to be able to detect changes within the sample. Blood smears were prepared, and two areas were marked on the slide using a grease pen; a smaller area (=control area) and a larger area (=test area). Both areas were treated identically unless otherwise described. Samples were washed twice using tris buffered saline (TBS; 0.05 M) prior to permeabilization of the RBCs using 0.1% trypsin. After another washing procedure, peroxidase activation was blocked using a methanol-hydrogen peroxide solution, followed by a third washing protocol. Unspecific binding sites were blocked using a 3% skim milk solution prior to application of the primary antibody to the test area only (one antibody per slide): Rabbit anti Human NOS III Serine1177 (1:500; Merck, Darmstadt, Germany, overnight at 4 °C) or anti-nitrotyrosine (1:500; Upstate/Millipore, Burlington, VT, USA, 1 h at room temperature). The control area was incubated using an antibody control solution. Slides were washed, unspecific binding sites were blocked using a 3% Normal Goat Serum, and samples were incubated with the secondary goat anti-rabbit antibody (1:150; Dako, Glostrup, Denmark). The staining was developed using 3,3-diaminobenzidine-tetrahydrochloride solution (Sigma-Aldrich, St. Louis, MO, USA) containing ammonium chloride, Nickel (II)-sulfate hexahydrate, ß-D-Glucose monohydrate, and glucose-oxidase. Slides were then dehydrated using increasing alcohol solutions, mounted using Entellan™ (Merck, Darmstadt, Germany), and covered.

Slides were placed in a transmitted-light microscope (Axiophot 1 microscope; Zeiss, Oberkochen, Germany), and a resolution of at least 200-fold was adjusted. The microscope was coupled to a camera (Progres Gryphax Prokyon; Jenoptik Optical Systems GmbH, Jena, Germany) and attached to a computer to take pictures of the stained RBCs and to process images taken. Staining intensity was expressed as gray values, which were analyzed using the software ImageJ 1.52a (National Institutes of Health, Bethesda, MD, USA). For staining intensity analysis, mean grey values were measured from the slide background, the control RBCs, and the RBCs of the test area. The net immunostaining intensity was calculated as previously described [13].

### 3.6. Statistical Analysis

R version 4.1.1 was used to analyze the data (R Core Team, Vienna, Austria, 2021). The change over time in blood parameters, aggregation parameters, and deformability was modeled with linear growth models using time as a polynomial predictor (linear, quadratic, cubic) and a random intercept. Due to the non-normality and heteroscedasticity of the residuals, robust procedures were applied (R package: robustlmm [30]). To assess whether there was a linear, quadratic or cubic change over time, models were compared using RMSE and conditional R^2^. Comparisons between the control group and patients were conducted using ANOVA with trimmed means (R package: WRS2; Mair, & Wilcox) and robust linear regression (R package: robust, [31] with time as categorical predictor comparing the control group and the patient group at each time point. An α-level of 0.050 (two-tailed) was considered significant.

GraphPad Prism software (Version 9.4, La Jolla, CA, USA) was used for visualizing the data. Due to blood volume restrictions, no measurements could be made for ferritin and CRP in Con.

## 4. Discussion

Since the beginning of the pandemic, the effective treatment for COVID-19 patients and its versatile underlying molecular actions are still under discussion. Rapid diagnosis and subsequent recovery without long-term adverse health effects are of paramount interest. Improved insight and a better understanding of rheological properties, as well as alterations in RBC morphology and activation and their effects on oxygen transport, may lead to new therapeutic strategies and could possibly improve the monitoring and evaluation of COVID-19 patients. Therefore, hemorheological assessment, in combination with hematological profiling, in particular, may be an asset in determining severe courses as well as a potential (pharmaceutical) target.

In the present study, we observed that besides an increased immunological and nitrosative stress response, both hematological and hemorheological variables were negatively affected in hospitalized COVID-19 patients compared to healthy individuals. Moreover, we showed that alterations in deformability and aggregation recovered during the course of the infection, respectively, within 30 days after hospitalization, whereas anemia may persist even longer.

RBC and its associated Hb, Hct, and MCHC values were already lower compared to control at hospital admission T0, which persisted during the whole study monitoring of 30 days. These results confirm existing evidence showing lower Hb, RBC count, higher ferritin, and RDW in severely ill COVID-19 patients [8]. Anemic tendencies are a common health problem in SARS-CoV-2 infected individuals, worsening with the severity of the disease [8,32]. The observed adversely affected RBC variables in aCOV even at admission time T0 shows that the blood composition is affected already due to the infection and not treatment-related. Assuming a mean SARS-CoV-2 incubation time of seven days [33] and a mean RBC maturation time of 7–14 days from bone marrow release to a functioning RBC [34], there are two possibilities for the alterations caused by the virus: First, the direct effect on the already circulating RBCs with detrimental effects on function (impaired deformability, increased aggregation) and aging (increased lysis/apoptosis seen by decreased RBCs, Hct, Hb) or second, a potentially negative effect on maturation that reduces the function of the matured RBCs after entering the circulation. The latter would explain the pronounced detrimental effects after T5 and T10, but that could also be due to severe treatment effects. On the other hand, if there is an impact of SARS-CoV-2 directly on circulating RBCs, newly synthesized and then normal functioning RBCs could explain the nearly reversed hemorheological values at T30 when the affected RBCs are mostly replaced. Kronstein-Wiedemann et al. showed that RBC precursors are a direct target of SARS-CoV-2, which might induce dysregulation in Hb- and iron-metabolism already during RBC maturation [35]. These observations suggest a possibly major contribution to the severity of the systemic course of COVID-19 but do not explain the immediate impact directly after infection. The increased observed nitrosative stress in aCOVs’ RBCs might be an explanation for induced cell stress with accelerated aging. After examining blood smears from hospitalized COVID-19 patients, Marchi et al. suggested enhanced immune-inflammatory stress hematopoiesis based on morphological alterations [26]. Labeling of RBCs in vivo may be an additional option to monitor the RBC state during RBC aging and infection and may explain the development of decreased RBC function [36].

Interestingly, RDW increased at the end of the study period, indicating a more diverse RBC population. Altered RDW in COVID-19 was already observed by other groups, associating elevated RDW with all-cause mortality [37] and RDW during hospitalization with a significantly increased risk of mortality and septic shock [8,38]. An increased RBC turnover rate by SARS-CoV-2 with accelerated lysis of old RBCs and synthesis of new cells would explain the observed reversed hemorheological alterations at T30 when the initially affected cells are gradually replaced.

In terms of RBC deformability, our results showed that aCOV patients had lower RBC deformability than Con, especially in the acute disease state (T2-T10). This is consistent with the findings of other groups, who identified lower RBC deformability in their studies [12,19]. In general, in other diseases like hereditary spherocytosis, diabetes mellitus, and sickle cell disease, deformability is used as a parameter for the severity or likelihood of microvascular complications [18,39,40] and may, therefore, also be used for virus-induced alterations. All RBC deformability parameters EI_max_, SS^1^/_2_, as well as the ratio of SS^1^/_2_/EI_max_—which is assumed to be a robust value in reflecting alterations of deformability when reporting and comparing various populations of RBCs during different clinical states [24]—showed decreased values in aCOV compared to Con. The enzyme RBC-NOS synthase produces the free radical and main vasodilator nitric oxide, which also contributes to improved deformability by binding to the RBCs’ cytoskeleton [13,14]. The RBC activation level was unaltered in aCOV and Con, which might suggest that the decreased deformability in aCOV is not compensated by a potential counterregulatory upregulation of NO. Measuring of RBCs’ NO content and bioavailability should be added to draw definite conclusions at this point.

A decrease in RBC morphology could be a further reason for lower deformability, as deformability is dependent on the shape and volume of the RBCs. Kubánková et al. reported that COVID-19 patients had significantly more heterogeneous size and deformation of RBCs than healthy controls, and Gérard et al. reported mushroom-shaped phenotypes [41,42]. In addition, we could previously show that even patients with mild COVID-19 symptoms after SARS-CoV-2 infection exhibited prolonged altered RBC morphology and rheological parameters [43]. As a result, a change in RBC morphology could be one factor contributing to decreased RBC deformability in COVID-19 patients, compromising RBCs’ oxygen-supply function. Furthermore, Thomas et al. found structural protein damage and membrane lipid remodeling in RBCs as probable causes of altered RBC morphology and decreased oxygen delivery during COVID-19 [44]. These alterations could be related to changes in cell physical properties, as plasma membrane composition interact with the cytoskeleton to affect the shape and size of RBCs, which is critical for microcirculatory flow [27,45]. There are several surface receptors where the SARS-CoV-2 can enter the cell [46] in RBCs, most notably via binding of membrane cluster of differentiation 147 (CD147) or Band-3 protein on the erythrocyte membrane, negatively affecting erythrocyte morphology and function [47,48]. This includes RBCs’ coping with oxidative stress [47] as well as hyperviscosity, hyperaggregation, and reduced deformability [48]. In this context, the binding of the SARS-CoV-2 Spike protein to Band-3 protein on the erythrocyte membrane was suggested to result in morphological abnormalities but also affect the release of oxygen to metabolically active tissues and COVID-19 worsening [47].

Interestingly, there was no difference in aCOV’s MCV compared to Con and no change in MCV over time. It is known that too high or too low MCV can negatively affect RBC deformability [25,49], but this does not seem to be the case in acute COVID-19. Instead, the SARS-CoV-2 spike protein seems to directly affect fibrinogen in the circulation, causing substantial impairment of fibrinolysis that may contribute to hypercoagulation observed in COVID-19-positive patients [50]. We also observed increased fibrinogen and D-Dimer concentrations in aCOV compared to Con with concomitant decreased values of deformability and increased aggregation [51]. Confirming this, Maier et al. reported hyperviscosity and associated thrombotic complications in COVID-19 patients [52]. The rise of blood viscosity could be a consequence of the rise of plasma fibrinogen concentration, as Nader et al. observed very high levels of RBC aggregation and increased fibrinogen levels [19].

Although the increased fibrinogen levels at hospital admission T0 gradually decreased over time, the inflammatory state during the infection could be one factor causing the observed fibrinogen levels and, thus, a potential source of RBC hyper-aggregation [17]. Virus-driven inflammation as an explanation is in line with our approximately six-fold increased ferritin and nine-fold increased CRP levels compared to clinical reference values [20,21], indicating a severe pro-inflammatory status in aCOV. While CRP is an established marker for acute inflammation and severity in COVID-19 and other diseases [53,54], ferritin can also be regarded as an acute phase protein besides its main function of iron storing and releasing [55]. Whereas CRP is decreasing over time in aCOV, ferritin levels are still elevated at T30, suggesting that it can be regarded as a longer-lasting marker for disease progression [56], subsequently negatively affecting hemorheological properties [57].

The assessment of leukocytes and neutrophils confirmed the activated immune system response in aCOV, showing highly increased values until T10. Conversely, the relative proportion of eosinophilic cells was highest at T30, suggesting an autoimmune reaction after one month. Although it was observed that eosinophilia reduced severity and symptoms in COVID-19-positive individuals [58], the observed increased values could, of course, result from the concomitant decreased neutrophil proportion. Roncati et al. found eosinophilia plus basophilia and degranulated eosinophils together with the rouleaux formation in peripheral blood smears from hospitalized COVID-19 patients just admitted to intensive care, representing cytological signals of a Th2 immune rather than an expected Th1 response [59]. Unfortunately, we did not have any additional FACS analysis to determine further immune cell subpopulations to draw a final conclusion regarding immune cell population alterations in aCOV.

In addition to this, increased D-Dimer concentrations in aCOV, already proposed as a biomarker for monitoring SARS-CoV-2 infected individuals [60], confirms apparently increased blood clotting in severe COVID-19 [3] together with concomitantly observed increased RBC aggregation. This may affect blood circulation in various ways: RBC aggregates may limit blood flow in the microcirculation and, therefore, oxygen supply to tissue and organs since RBCs are only able to pass through small vessels as single cells and not as aggregates [61]. The formation of clots and clot stability also are affected by increased RBC aggregation [19]. Due to the enhanced RBC aggregation and the resulting increased blood viscosity, COVID-19 patients are at greater risk for thrombosis [23], as evidenced by the high prevalence of thrombotic events and other vascular complications among hospitalized patients [62,63]. All these mechanisms could be contributors to the disease severity in COVID-19 patients caused by increased RBC aggregation.

The shortening of aggregation half-time T^1^/_2_, which represents the kinetics of the aggregation, indicates that aCOV RBC aggregates form rouleaux at a shorter time after their disaggregation compared to Con. This result reflects a higher tendency for aggregation among aCOV, especially after the incubation period with SARS-CoV-2. The shear rate at disc min represents the minimal shear rate needed to prevent RBC aggregation. An elevated shear rate indicates a higher tendency for aggregation and aggregate stability in aCOV over the first ten days. This implies that an increased shear rate was needed for complete disaggregation in these SARS-CoV-2 infected individuals, which, in turn, possibly contributes to blood clotting and elevated thrombosis risk frequently observed in COVID-19 [63,64].

Our findings show that initially increased aggregation index (AI) values of COVID-19 patients decreased over the duration of the study, particularly from T0 to T5. Reduced aggregation is the result of lower AI, which is advantageous for patients [22]. The definite reasons for the decrease over time can only be assumed. The medication used for thrombosis prophylaxis could be one reason for the decrease in AI. Anticoagulation therapy is commonly used in COVID-19 patients to prevent thrombotic events or to treat acquired thrombosis [65]. For high D-dimer values, prophylactic anticoagulation with low-molecular-weight heparin (LMWH) has been considered, which might influence RBC aggregation [65]. Furthermore, anti-inflammatory drugs are used to inhibit the immune response in COVID-19 patients [1]. According to emerging data, an altered inflammatory response and excessive release of pro-inflammatory cytokines such as interleukin-6, interferon-gamma, and tumor necrosis factor-alpha are important components of the virus´ induced damage [1]. As previously stated, the inflammatory state also influences fibrinogen plasma levels, which contributes to RBC aggregation. As a result, if the immune system is suppressed due to the anti-inflammatory medication, RBC aggregation may also be reduced. It has been acknowledged that severe COVID-19 is characterized not only by systemic hyperinflammation and coagulopathy but also by T-cell deficiencies [66]. In this context, the suppression of the immune system acts as a double-edged sword, while the development of novel balanced immunomodulatory approaches, combining both suppressive and activating immunotherapies, along with LMWH in selected cases, may warrant a better treatment strategy for severe COVID-19 patients [67,68].

Additionally, altered RBC structural proteins may play a role in the thromboembolic and coagulopathic issues faced by certain critically ill patients [44]. Even after recovery, individuals showed altered phenotypes and significant pathological abnormalities in microcirculation, though not to the extent that they were during the acute phase of infection [42,69]. As a result, this subject may be significant in the context of Long-COVID. However, there is no evidence to support this in the case of Long-COVID, so further research is needed.

### 4.1. Further Considerations and Other Factors

In addition to mechanical stress, oxidative stress may have an impact on RBCs as well. Increased RBC intracellular reactive oxygen/nitrogen species (iROS) levels have been shown to reduce RBC deformability in sepsis: a similar mechanism could possibly reduce deformability in COVID-19 as a result of increased oxidative stress [57]. It has been demonstrated that oxidative damage to the RBC membrane has a considerable impact on the membrane’s viscoelastic characteristics and, thus, on its deformability, e.g., in sickle cell anemia [70,71]. We observed increased nitrotyrosine staining in aCOV at hospital admission compared to Con, which is a sign of the potentially high oxidative stress in RBCs causing intracellular damage and reducing RBCs’ life span. This is in line with observations that COVID-19 can potentially cause the most severe destruction to RBCs in eryptosis, the final stage of RBC cell death, caused by persistent hyperinflammation and exposure to oxidative stress [57]. Thus, the assessment of the altered stress response of SARS-CoV-2 infected RBCs may be a valuable diagnostic addition in future studies.

Beside hemorheological assessment, other cell-based prognostic markers are under examination or already established in COVID-19 inflammation monitoring. Both increased monocyte distribution width (MDW) [72,73] and higher neutrophil-to-lymphocyte ratios (NLR) [74,75] predicted severity and mortality in COVID-19 patients and can easily be obtained during hematological measurements. Thus, for the evaluation of the severity of the disease, it would be useful to use both cell-based systems markers as a combined approach, as there are many indications that both systems are influenced by SARS-CoV2 and that different results regarding the markers can be expected in different phases of the disease. While standard procedures focus mainly on inflammatory markers, RBC parameters should not be neglected.

As a result, therapeutic approaches to reduce oxidative stress and immune response may be capable of minimizing RBC damage during COVID-19 infection and may result in better RBC deformability and improved oxygen supply. Downregulation of the immune response and therapeutic medications could also affect the potential regeneration of RBC deformability with subsequent reduction of persistent symptoms. 

### 4.2. Limitations

There are some limitations that have to be mentioned. One limitation is the small number of participants in the study population and the reduction in sample size over time when patients had already left the hospital before T10 or T30, which resulted in partly incomplete data sets of blood samples and associated measures for each patient. In addition, admission of clinically ill patients at night led to the necessity to collect and measure hemorheological and hematological variables after some hours of blood storing at 4 °C resulting in variability in duration from collection to measurement. However, results suggest that this factor was unlikely to cause any discrepancies in the measured values. Vaccination status could not be obtained in the majority of aCOV due to language skills or rapid inpatient release/death, whereas all individuals in CON were vaccinated. The possible influence of vaccination before hospital admission and concomitant disease severity with regard to the present parameters have still been determined with no possible conclusion at the moment.

## 5. Conclusions

Due to their significant impact on microcirculation and oxygen supply, rheological properties such as RBC deformability and aggregation could play a significant role in the treatment of COVID-19 and the possible prevention of severe Long-/Post-COVID cases.

We observed that RBC hematological and hemorheological variables are pathologically affected in hospitalized COVID-19 patients compared to healthy controls especially impaired during acute infection. Because of their close relationship with the immune response and intracellular stress, they may be sufficient indicators for severe COVID-19 cases and may provide another additional asset to monitor COVID-19 severity and therapy in combination with hematological assessment. Both deformability and aggregation appear to be usable for appropriate severity parameters because they are, on the one hand, validly measurable, with moderate material and time requirements, and, on the other hand, directly influenced by the effects of COVID-19 infection. In addition, examining RBC deformability and aggregation as well as ROS/RNS stress induction in relation to both vaccination status and patient outcome, as well as to any long-term COVID symptoms, may be beneficial in addition to established inflammatory parameters like CRP or ferritin.

## Figures and Tables

**Table 1:** General characteristics of acute infected and severely ill COVID-19 patients (aCOV) and an age-matched healthy control group (Con). All data are presented as mean ± Standard Deviation.

	aCOV		Con	
**Age [years]**	59.0 ± 16.9		52.1 ± 18.1	
**Gender distribution**	Female	n = 19	Female	n = 24
	Male	n = 25	Male	n = 20
**Vaccination status**	Vaccinated	n = 12	Vaccinated	n = 44
	Not vaccinated	n = 7		
	Unknown	n = 25		
**Inpatient stay**	Infectious ward	n = 33		
	Intensive care unit	n = 11		

## Data Availability

All data which are not provided in the main manuscript or Supplementary files can be provided by the corresponding author upon reasonable request. Not all data are publicly available due to privacy restrictions.

## Supplementary Materials

The following supporting information can be downloaded at: https://www.mdpi.com/article/10.3390/ijms232315332/s1, Supplementary Materials File S1: Detailed descriptive statistics + statistical data for all determined variables; Table S1: Descriptive Statistics and statistical results of hematological variables; Table S2: Descriptive Statistics and statistical results of hemorheological variables; Table S3: Descriptive Statistics and statistical results of erythrocyte staining intensities. Supplementary references: [30].
