# Supplementary material for: SARS-CoV-2 Altered Hemorheological and Hematological Parameters during One-Month Observation Period in Critically Ill COVID-19 Patients
Source: Int J Mol Sci. 2022 Dec 5;23(23):15332. doi: 10.3390/ijms232315332 (PMC9735540; doi:10.3390/ijms232315332)
Supplement: Supplementary file 1 [file ijms-23-15332-s001.zip › ijms-2058103-supplementary.pdf]

Supplemental S1: Detailed descriptive statistics + statistical data for all determined variables

Table S1

Descriptive Statistics and statistical results of hematological variables

	CON		aCOV T0		aCOV T2		aCOV T5		aCOV T10		aCOV T30	
	M	SD	M	SD	M	SD	M	SD	M	SD	M	SD
**Neutrophil Granulocytes [10^9/L]**	57.02	14.86	74.91	12.18	78.28	8.05	73.97	10.69	78.06	9.50	58.76	16.00
**Ferritin [µg/L]**	-	-	1555.36	3159.39	1139.44	948.76	966.72	852.90	1132.22	1425.73	1789.17	2062.31
**D-Dimers [mg/IU]**	1.03	3.54	2.33	4.95	3.46	6.36	5.83	7.81	4.03	5.50	1.89	2.51
**Erythrocytes [10^{12}/L]**	4.49	0.63	4.06	0.72	3.77	0.69	4.10	0.74	3.81	0.67	3.90	0.80
**Leukocytes [10^9/L]**	6.21	2.72	7.88	9.75	6.94	3.12	8.36	3.04	10.50	3.90	10.92	14.71
**MCV [fl]**	90.22	5.76	90.18	6.76	90.36	7.96	88.25	6.83	90.75	4.97	93.55	6.54
**MCHC [g/dL]**	34.39	1.25	34.07	0.93	33.60	1.17	33.96	0.97	33.67	0.56	33.30	0.99
**Hematocrit [%]**	40.00	5.00	36.00	6.00	34.00	6.00	36.00	6.00	35.00	7.00	36.00	7.00
**Hemoglobin [g/dL]**	13.89	1.75	12.39	2.00	11.38	1.95	12.25	2.24	11.70	2.45	12.08	2.28

**MCH [pg]**	31.05	2.62	30.73	2.45	30.33	2.65	29.96	2.33	30.57	1.86	31.15	2.41
**Thrombocytes [10^9/L]**	236.36	63.16	192.17	76.93	221.56	75.78	283.97	123.16	207.45	93.58	235.19	75.26
**Relative Eosinophils [%]**	2.08	1.78	0.30	0.68	0.75	1.24	1.09	1.23	0.70	0.72	2.89	2.08
**CRP [mg/L]**	-	-	95.52	71.70	70.04	64.25	19.89	21.34	23.12	32.26	19.31	25.43
**Fibrinogen [g/L]**	3.45	0.93	4.51	1.19	4.26	1.77	3.06	1.58	4.10	1.97	3.36	1.48
**RDW [%]**	13.37	1.30	13.87	1.37	14.06	1.50	13.67	2.44	15.18	3.35	15.26	2.46

Modeling of time courses: blood parameters

Model performance: robust lmer

		$R^2_{conditional}$	$R^2_{marginal}$	ICC	RMSE	σ
**Neutrophil Granulocytes**	M0	0.35	0.00	0.35	9.3	9.75
	**linear**	0.49	0.2	0.35	8.64	8.87
	quadratic	0.46	0.23	0.3	8.91	9.12
	cubic	0.45	0.23	0.29	8.97	9.22
**Ferritin**	M0	0.91	0.00	0.91	1334.69	250.83
	linear	0.92	0.00	0.92	1332.26	237.67
	**quadratic**	0.92	0.01	0.92	1313.69	236.43
	cubic	0.92	0.01	0.92	1314.07	239.62
**D-Dimers**	M0	0.71	0.00	0.71	3.36	0.6
	linear	0.7	0.00	0.7	3.36	0.6
	quadratic	0.7	0.00	0.7	3.36	0.6
	**cubic**	0.67	0.05	0.65	3.33	0.67
**Erythrocytes**	M0	0.76	0.00	0.76	0.33	0.35
	**linear**	0.75	0.00	0.75	0.32	0.36
	quadratic	0.76	0.01	0.75	0.32	0.36
	cubic	0.76	0.01	0.76	0.32	0.35
**Leukocytes**	M0	0.21	0.00	0.21	2.68	2.79
	linear	0.25	0.02	0.24	2.62	2.7
	**quadratic**	0.43	0.11	0.36	2.26	2.39
	cubic	0.42	0.11	0.35	2.26	2.42
**MCV**	M0	0.93	0.00	0.93	1.95	1.6
	linear	0.93	0.01	0.93	1.88	1.62
	**quadratic**	0.93	0.01	0.93	1.86	1.67
	cubic	0.93	0.01	0.93	1.87	1.62

**MCHC**	M0	0.63	0.00	0.63	0.63	0.59
	linear	0.64	0.03	0.63	0.62	0.57
	quadratic	0.65	0.03	0.64	0.61	0.57
	**cubic**	0.66	0.03	0.65	0.6	0.57
**Hematocrit**	M0	0.73	0.00	0.73	0.03	0.03
	linear	0.72	0.00	0.72	0.03	0.03
	quadratic	0.72	0.00	0.72	0.03	0.03
	**cubic**	0.73	0	0.72	0.03	0.03
**Hemoglobin**	M0	0.78	0.00	0.78	0.9	0.93
	**linea**r	0.77	0.00	0.77	0.89	0.94
	quadratic	0.77	0.00	0.77	0.89	0.95
	cubic	0.77	0.01	0.77	0.89	0.96
**MCH**	M0	0.93	0.00	0.93	0.68	0.59
	linear	0.94	0.00	0.94	0.68	0.58
	quadratic	0.94	0.00	0.94	0.65	0.59
	**cubic**	0.95	0.01	0.95	0.64	0.52
**Thrombocytes**	M0	0.24	0.0	0.24	75.53	77.59
	linear	0.37	0.03	0.35	69	69.45
	quadratic	0.43	0.07	0.38	65.15	66.22
	**cubic**	0.51	0.13	0.43	57.59	62.67
**Relative Eosinophils**	M0	0	0.00	0.00	1.44	0.93
	linear	0.54	0.54	0.00	1.17	0.76
	quadratic	0.55	0.55	0.00	1.17	0.76
	**cubic**	0.55	0.55	0	1.15	0.78
**CRP**	M0	0	0.00	0.00	63.18	50.04
	linear	0.25	0.11	0.15	53.2	45.82

	quadratic	0.59	0.28	0.43	40.67	37.11
	**cubic**	0.63	0.32	0.46	39.6	34.91
**Fibrinogen**	M0	0.38	0.00	0.38	1.02	1.17
	linear	0.43	0.03	0.41	0.98	1.14
	quadratic	0.62	0.12	0.56	0.85	0.92
	**cubic**	0.62	0.16	0.55	0.76	0.92
**RDW**	M0	0.69	0.00	0.69	1.42	0.77
	linear	0.69	0.04	0.68	1.36	0.78
	quadratic	0.7	0.04	0.69	1.36	0.77
	**cubic**	0.76	0.07	0.74	1.33	0.69

Note. Model fit robust mixed effects regression models. M0: Intercept-only-Model

Modeling over time

		Model 1				***Model 2***				***Model 3***			
		b	**SE**	***t***	***p***	***b***	**SE**	***t***	***p***	***b***	**SE**	***t***	***p***
**Neutrophil Granulocytes**	intercept	76.83	1.52	5.41	**<.001**	76.14	1.64	46.46	< .001	76.29	1.73	44.17	<.001
	time	-.57	.10	-5.70	**<.001**	-0.04	0.47	-0.09	.925	-0.33	1.17	-0.28	.782
	time²					-0.02	0.02	-1.19	.238	0.03	0.17	0.16	.871
	time³										0.00	-0.27	.786
**Ferritin**	intercept	927.16	139.50	6.65	<.001	989.97	145.14	6.82	**< .001**	1007.19	149.96	6.72	<.001
	time	-4.55	4.18	-1.09	.280	-31.63	12.93	-2.45	**<.050**	-46.87	35.22	-1.33	.188
	time²					1.08	0.44	2.47	**<.050**	3.33	4.61	0.72	.472
	time³									-0.06	0.12	-0.50	.620

**D-Dimers**	intercept	1.12	.16	6.99	<.001	1.00	0.17	6.00	< .001	0.98	0.17	5.65	**<.001**
	time	.00	.01	.48	.631	0.10	0.03	3.49	< .001	0.15	0.08	1.89	**.062**
	$time^2$					0.00	0.00	-3.52	< .001	-0.01	0.01	-0.97	**.335**
	$time^3$									0.00	0.00	0.64	**.522**
**Erythrocytes**	intercept	4.10	.11	39.03	**<.001**	4.09	0.11	37.69	< .001	4.06	0.11	36.84	<.001
	time	-.01	.00	-1.38	**.172**	0.00	0.02	0.24	.808	0.06	0.04	1.35	.181
	$time^2$					0.00	0.00	-0.56	.577	-0.01	0.01	-1.42	.158
	$time^3$									0.00	0.00	1.38	.171
**Leukocytes**	intercept	7.05	.39	18.15	<.001	6.34	0.43	14.65	**< .001**	6.40	0.46	14.07	<.001
	time	.05	.03	1.73	.088	0.48	0.11	4.39	**< .001**	0.37	0.27	1.36	.179
	$time^2$					-0.01	0.00	-4.08	**< .001**	0.00	0.04	0.10	.919
	$time^3$									0.00	0.00	-0.47	.639
**MCV**	intercept	89.68	.96	93.88	<.001	89.83	0.96	93.38	**< .001**	89.94	0.96	93.27	< .001
	time	.05	.02	2.96	.004	-0.04	0.08	-0.50	**.622**	-0.28	0.19	-1.48	.143
	$time^2$					0.00	0.00	1.17	**.245**	0.04	0.03	1.52	.133
	$time^3$									0.00	0.00	-1.42	.160
**MCHC**	intercept	33.90	.13	254.20	<.001	33.96	0.14	238.87	< .001	33.98	0.15	232.48	**<.001**
	time	-.02	.01	-2.73	.008	-0.05	0.03	-1.81	.074	-0.10	0.07	-1.57	**.119**
	$time^2$					0.00	0.00	1.27	.209	0.01	0.01	1.00	**.321**
	$time^3$									0.00	0.00	-0.88	**.382**
**Hematocrit**	intercept	.37	.01	44.07	<.001	0.37	0.01	42.42	< .001	0.37	0.01	41.17	**< .001**
	time	.00	.00	-.55	.584	0.00	0.00	0.09	.929	0.00	0.00	1.09	**.278**
	$time^2$					0.00	0.00	-0.22	.830	0.00	0.00	-1.17	**.246**
	$time^3$									0.00	0.00	1.15	**.252**
**Hemoglobin**	intercept	12.53	.29	43.08	**<.001**	12.53	0.30	41.81	< .001	12.48	0.31	40.83	<.001
	time	-.01	.01	-1.15	**.253**	-0.01	0.05	-0.14	.891	0.08	0.11	0.71	.482
	$time^2$					0.00	0.00	-0.12	.908	-0.01	0.02	-0.86	.394

	$time^3$									0.00	0.00	0.85	.396
**MCH**	intercept	3.35	.35	86.44	<.001	30.45	0.36	85.62	< .001	30.51	0.36	85.91	**<.001**
	time	.01	.01	.97	.335	-0.05	0.03	-1.90	.061	-0.20	0.06	-3.36	**.001**
	$time^2$					0.00	0.00	2.18	<.050	0.03	0.01	2.97	**.004**
	$time^3$									0.00	0.00	-2.77	**.007**
**Thrombocytes**	intercept	208.54	11.35	18.38	<.001	195.82	12.28	15.94	< .001	182.29	12.65	14.41	**<.001**
	time	1.47	.69	2.11	.038	9.37	3.07	3.05	< .010	34.71	7.11	4.88	**<.001**
	$time^2$					-0.26	0.10	-2.61	<. 050	-4.33	1.04	-4.16	**<.001**
	$time^3$									0.11	0.03	3.93	**<.001**
**Relative Eosinophils**	intercept	.25	.09	2.74	.008	0.28	0.11	2.49	.015	0.21	0.12	1.68	**.098**
	time	.09	.01	1.54	<.001	0.07	0.04	1.86	.067	0.22	0.09	2.38	**.020**
	$time^2$					0.00	0.00	0.48	.630	-0.02	0.01	-1.71	**.091**
	$time^3$									0.00	0.00	1.75	**.084**
**CRP**	intercept	59.63	5.58	1.69	<.001	83.03	6.91	12.02	< .001	91.23	7.12	12.82	**<.001**
	time	-2.50	.56	-4.45	<.001	-12.51	1.41	-8.85	< .001	-21.74	3.38	-6.42	**<.001**
	$time^2$					0.34	0.05	7.15	< .001	1.67	0.46	3.64	**<.001**
	$time^3$									-0.03	0.01	-2.90	**.005**
**Fibrinogen**	intercept	4.03	.31	13.01	<.001	4.44	0.33	13.33	< .001	4.78	0.36	13.34	**<.001**
	time	-.03	.02	-1.47	.150	-0.23	0.07	-3.41	<.010	-0.61	0.17	-3.59	**.001**
	$time^2$					0.01	0.00	3.09	<.010	0.06	0.02	2.79	**.009**
	$time^3$									0.00	0.00	-2.53	**.016**
**RDW**	intercept	13.63	.20	68.34	<.001	13.68	0.21	66.22	< .001	13.79	0.21	66.20	**<.001**
	time	.03	.01	3.34	.001	0.00	0.04	-0.10	.917	-0.23	0.08	-2.85	**.005**
	$time^2$					0.00	0.00	0.86	.392	0.04	0.01	3.28	**.002**
	$time^3$									0.00	0.00	-3.23	**.002**

Note. Parameter estimation by robust linear mixed effects regression model (Koller, M. robustlmm: An R Package for Robust Estimation of Linear Mixed-Effects Models. J. Stat. Softw. 2016, 75, 1–24. https://doi.org/10.18637/jss.v075.i06.). Model 1 linear prediction of concentration of blood parameter by time (0, 2, 5, 10, 30). Model quadratic prediction of concentration of blood parameters by time (0, 2, 5, 10, 30). Model of cubic prediction of concentration of blood parameters by time (0, 2, 5, 10, 30).

Blood parameters: CON vs. EG

Overall statistics: sign. result suggests a significant difference between trimmed means across control group and different time points (aCOV)

**Neutrophils**	$F(5, 26.46) = 9.72$, $\xi = 0.67$ [0.47; 0.87]	$p < .001$
**Erythrocytes**	$F(5, 27.85) = 3.09$, $\xi = 0.45$ [0.25; 0.64]	$p < .050$
**Leukocytes**	$F(5, 28.41) = 2.87$, $\xi = 0.55$ [0.31; 0.92]	$p < .050$
**MCV**	$F(5, 28.39) = 0.941$, $\xi = 0.50$ [0.18; 0.68]	$p = .470$
**MCHC**	$F(5, 29.03) = 3.32$, $\xi = 0.53$ [0.29; 0.77]	$p <.050$
**Hemoglobin**	$F(5, 27.6) = 6.08$, $\xi = 0.5$ [0.28; 0.67]	$p <.001$
**Hematocrit**	$F(5, 27.87) = 3.87$, $\xi = 0.47$ [0.27; 0.67]	$p <.010$
**MCH**	$F(5, 28.56) = 1.11$, $\xi = 0.38$ [0.19; 0.6]	$p = .376$
**Thrombocytes**	$F(5, 8.89) = 3.92$, $\xi = 0.47$ [0.22; 0.78]	$p < .010$
**Eosinophils**	$F(5, 21.43) = 15.38$, $\xi = 0.7$ [0.45; 1.09]	$p < .001$
**RDW**	$F(5, 27.74) = 2.55$, $\xi = 0.53$ [0.27; 0.91]	$p = .051$,
**Fibrinogen**	$F(5, 12.61) = 2.55$, $\xi = 0.61$ [0.34; 0.99]	$p = .083$
**D-Dimers**	$F(5, 24.19) = 4.06$, $\xi = 1.06$ [0.39;3],	$p <.010$

(https://doi.org/10.3758/s13428-019-01246-w; effect size= ξ [95%-bootstrap CI]: 0.1 small, 0.3 medium, 0.5 large). Overall statistic calculated using WRS2 (Mair, & Wilcox)

		b	*SE*	95%-CI-low	95%-CI-high	*t*	df_{error}	*p*
**Neutrophil Granulocytes**	(Intercept)	59.23	2.24	54.79	63.67	26.39	127	<.001
	CON_vs_aCOV T0	17.22	3.02	11.25	23.20	5.70	127	<.001
	CON_vs_aCOV T2	19.05	3.66	11.80	26.30	5.20	127	<.001
	CON_vs_aCOV T5	14.74	3.60	7.61	21.87	4.09	127	<.001
	CON_vs_aCOV T10	18.83	5.15	8.63	29.03	3.65	127	<.001
	CON_vs_aCOV T30	-3.31	4.37	-11.96	5.33	-0.76	127	.45
**Erythrocytes**	(Intercept)	4.49	0.12	4.26	4.73	37.47	147	<.001
	CON_vs_aCOV T0	-0.38	0.16	-0.70	-0.07	-2.40	147	.02
	CON_vs_aCOV T2	-0.72	0.19	-1.10	-0.35	-3.79	147	<.001
	CON_vs_aCOV T5	-0.39	0.18	-0.75	-0.04	-2.20	147	.03
	CON_vs_aCOV T10	-0.68	0.26	-1.19	-0.18	-2.66	147	.01
	CON_vs_aCOV T30	-0.60	0.21	-1.01	-0.18	-2.86	147	<.001
**Leukocytes**	(Intercept)	6.10	0.53	5.05	7.15	11.52	147	<.001
	CON_vs_aCOV T0	0.10	0.71	-1.30	1.50	0.14	147	.89
	CON_vs_aCOV T2	0.42	0.85	-1.26	2.09	0.49	147	.62
	CON_vs_aCOV T5	2.25	0.79	0.70	3.81	2.86	147	<.001
	CON_vs_aCOV T10	3.52	1.17	1.20	5.83	3.00	147	<.001
	CON_vs_aCOV T30	1.28	0.93	-0.55	3.12	1.38	147	.17
**MCV**	(Intercept)	89.94	1.31	87.36	92.53	68.69	147	<.001
	CON_vs_aCOV T0	-0.48	1.74	-3.93	2.96	-0.28	147	.78
	CON_vs_aCOV T2	-1.38	2.14	-5.61	2.85	-0.65	147	.52
	CON_vs_aCOV T5	-2.08	1.96	-5.96	1.80	-1.06	147	.29
	CON_vs_aCOV T10	0.81	2.78	-4.68	6.29	0.29	147	.77
	CON_vs_aCOV T30	3.44	2.32	-1.15	8.03	1.48	147	.14
**MCHC**	(Intercept)	34.60	0.18	34.24	34.96	189.62	147	<.001
	CON_vs_aCOV T0	-0.62	0.24	-1.09	-0.15	-2.59	147	.01
	CON_vs_aCOV T2	-0.96	0.29	-1.53	-0.39	-3.31	147	<.001

	CON_vs_aCOV T5	-0.63	0.27	-1.16	-0.11	-2.37	147	.02
	CON_vs_aCOV T10	-0.93	0.38	-1.68	-0.18	-2.46	147	.02
	CON_vs_aCOV T30	-1.31	0.32	-1.94	-0.69	-4.16	147	<.001
**Hematocrit**	(Intercept)	0.40	0.01	0.38	0.43	34.88	147	<.001
	CON_vs_aCOV T0	-0.03	0.02	-0.07	0.00	-2.25	147	.03
	CON_vs_aCOV T2	-0.06	0.02	-0.10	-0.03	-3.49	147	<.001
	CON_vs_aCOV T5	-0.04	0.02	-0.08	-0.01	-2.50	147	.01
	CON_vs_aCOV T10	-0.06	0.02	-0.11	-0.01	-2.24	147	.03
	CON_vs_aCOV T30	-0.04	0.02	-0.08	0.00	-1.85	147	.07
**Hemoglobin**	(Intercept)	13.97	0.40	13.17	14.77	34.51	147	<.001
	CON_vs_aCOV T0	-1.41	0.54	-2.47	-0.35	-2.64	147	.01
	CON_vs_aCOV T2	-2.59	0.64	-3.86	-1.32	-4.03	147	<.001
	CON_vs_aCOV T5	-1.71	0.60	-2.91	-0.52	-2.83	147	.01
	CON_vs_aCOV T10	-2.27	0.87	-3.98	-0.56	-2.62	147	.01
	CON_vs_aCOV T30	-1.83	0.71	-3.23	-0.43	-2.58	147	.01
**MCH**	(Intercept)	31.04	0.46	30.14	31.95	67.93	147	<.001
	CON_vs_aCOV T0	-0.49	0.60	-1.67	0.69	-0.82	147	.42
	CON_vs_aCOV T2	-1.04	0.72	-2.46	0.37	-1.45	147	.15
	CON_vs_aCOV T5	-1.09	0.67	-2.41	0.23	-1.63	147	.10
	CON_vs_aCOV T10	-0.47	0.94	-2.34	1.39	-0.50	147	.62
	CON_vs_aCOV T30	0.04	0.78	-1.49	1.58	0.06	147	.96
**Thrombocytes**	(Intercept)	236.14	17.01	202.51	269.76	13.88	147	<.001
	CON_vs_aCOV T0	-48.79	22.61	-93.47	-4.10	-2.16	147	.03
	CON_vs_aCOV T2	-14.83	27.07	-68.33	38.66	-0.55	147	.58
	CON_vs_aCOV T5	14.22	26.44	-38.03	66.46	0.54	147	.59
	CON_vs_aCOV T10	-29.58	36.38	-101.47	42.32	-0.81	147	.42
	CON_vs_aCOV T30	-0.22	29.77	-59.05	58.61	-0.01	147	.99
**Relative Eosinophils**	(Intercept)	1.65	0.15	1.35	1.95	10.90	127	<.001

	CON_vs_aCOV T0	-1.44	0.20	-1.84	-1.05	-7.17	127	<.001
	CON_vs_aCOV T2	-1.39	0.26	-1.91	-0.87	-5.30	127	<.001
	CON_vs_aCOV T5	-0.89	0.24	-1.37	-0.40	-3.64	127	<.001
	CON_vs_aCOV T10	-0.96	0.33	-1.62	-0.30	-2.87	127	<.001
	CON_vs_aCOV T30	1.23	0.35	0.53	1.93	3.47	127	<.001
**RDW**	(Intercept)	13.23	0.22	12.79	13.66	60.21	147	<.001
	CON_vs_aCOV T0	0.49	0.29	-0.09	1.06	1.68	147	.09
	CON_vs_aCOV T2	0.52	0.35	-0.18	1.21	1.47	147	.14
	CON_vs_aCOV T5	0.01	0.33	-0.64	0.67	0.04	147	.97
	CON_vs_aCOV T10	0.99	0.48	0.05	1.94	2.08	147	.04
	CON_vs_aCOV T30	1.33	0.39	0.56	2.09	3.41	147	<.001
**Fibrinogen**	(Intercept)	3.30	0.29	2.74	3.87	11.58	79	<.001
	CON_vs_aCOV T0	1.20	0.56	0.08	2.32	2.14	79	.04
	CON_vs_aCOV T2	0.56	0.55	-0.54	1.65	1.01	79	.31
	CON_vs_aCOV T5	-0.63	0.57	-1.77	0.51	-1.10	79	.27
	CON_vs_aCOV T10	0.73	0.92	-1.10	2.56	0.79	79	.43
	CON_vs_aCOV T30	0.45	0.74	-1.01	1.92	0.62	79	.54
**D-Dimers**	(Intercept)	0.42	0.14	0.14	0.70	2.98	145	<.001
	CON_vs_aCOV T0	0.33	0.19	-0.05	0.71	1.73	145	.09
	CON_vs_aCOV T2	0.37	0.22	-0.06	0.80	1.70	145	.09
	CON_vs_aCOV T5	0.65	0.26	0.13	1.16	2.49	145	.01
	CON_vs_aCOV T10	0.87	0.32	0.23	1.50	2.69	145	.01
	CON_vs_aCOV T30	0.62	0.35	-0.07	1.31	1.79	145	.08

Table S2

Descriptive Statistics and statistical results of hemorheological variables

	CON		aCOV T0		aCOV T2		aCOV T5		aCOV T10		aCOV T30	
	M	SD	M	SD	M	SD	M	SD	M	SD	M	SD
**EI max [au]**	0.50	0.02	0.49	0.02	0.48	0.03	0.47	0.03	0.47	0.04	0.49	0.03
**SS ½**	1.23	0.20	1.15	0.23	1.09	0.19	1.07	0.21	1.12	0.27	1.13	0.19
**SS ½ EI max**	2.47	0.34	2.36	0.43	2.27	0.32	2.25	0.36	2.33	0.47	2.31	0.30
**Isc max**	34.25	5.69	34.71	6.63	35.57	7.24	37.72	5.39	34.51	4.29	36.07	4.46
**AI [%]**	59.01	7.41	65.83	7.27	62.46	11.21	58.25	11.98	59.21	11.15	59.47	8.37
**Shr at dIsc min**	154.83	152.91	326.69	206.78	260.13	115.41	258.60	210.55	246.88	159.18	173.33	89.30
**T ½ ($^1/_s$)**	2.77	1.02	1.92	0.77	2.44	1.35	2.99	1.98	2.96	1.65	2.73	1.11

Time differences- Time as factor

Deformability

EI max

F(5, 36.79) = 4.38, p <.001, ES = 0.42 [0.23, 0.63]

	b	***SE***	**95 % - CI**	**95 %- CI**	***t***	**df $_{error}$**	***p***
**(Intercept)**	0.5	0	0.49	0.5	124.25	184	<.001
**CON_vs_aCOV T0**	-0.01	0.01	-0.02	0	-1.47	184	.145
**CON_vs_aCOV T2**	-0.02	0.01	-0.03	0	-2.78	184	.006
**CON_vs_aCOV T5**	-0.02	0.01	-0.04	-0.01	-3.86	184	<.001
**CON_vs_aCOV T10**	-0.02	0.01	-0.04	-0.01	-3.14	184	.002
**CON_vs_aCOV T30**	-0.01	0.01	-0.03	0.01	-1.01	184	.315

SS ½

F (5, 38.3) = 2.36, p = .059, ES = 0.38 [0.18; 0.6]

	b	*SE*	95 % - CI	95 %- CI	*t*	df $_{error}$	*p*
**(Intercept)**	1.23	0.03	1.16	1.29	37.88	184	<.001
**CON_vs_aCOV T0**	-0.08	0.05	-0.17	0.02	-1.61	184	.110
**CON_vs_aCOV T2**	-0.13	0.05	-0.23	-0.04	-2.85	184	.005
**CON_vs_aCOV T5**	-0.16	0.05	-0.25	-0.06	-3.2	184	.002
**CON_vs_aCOV T10**	-0.11	0.06	-0.22	0	-1.92	184	.056
**CON_vs_aCOV T30**	-0.1	0.07	-0.23	0.04	-1.37	184	.173

SS ½ / EI max Ratio

F(5, 38.51) = 1.55, p= .199, ES = 0.36 [0.16; 0.6]

	b	*SE*	95 % - CI	95 %- CI	*t*	df $_{error}$	*p*
**(Intercept)**	2.47	0.06	2.36	2.58	43.86	184	<.001
**CON_vs_aCOV T0**	-0.11	0.08	-0.27	0.05	-1.35	184	.177
**CON_vs_aCOV T2**	-0.2	0.08	-0.36	-0.04	-2.44	184	.016
**CON_vs_aCOV T5**	-0.22	0.09	-0.39	-0.05	-2.55	184	.012
**CON_vs_aCOV T10**	-0.14	0.1	-0.33	0.06	-1.38	184	.171
**CON_vs_aCOV T30**	-0.16	0.12	-0.4	0.08	-1.29	184	.197

Robust Models: Changes over time

	b	SE	*t*	*p*	R^2 cond. [marginal]	*RMSE*

**EI max**	(Intercept)	0.48664	0.00350	138.99710	.00000	0.363 [0.031]	0.020
	time	-0.00190	0.00076	-2.48783	.01431		
	time²	0.00006	0.00003	2.41461	.01734		
**SS ½**	(Intercept)	1.11130	0.02669	41.64450	.00000	0.375 [0.009]	0.152
	time	-0.00637	0.00570	-1.11842	.26579		
	time²	0.00024	0.00019	1.29852	.19676		
**SS ½ EI max**	(Intercept)	2.28658	0.04548	50.27441	.00000	0.416 [0.004]	0.256
	time	-0.00553	0.00931	-0.59353	.55404		
	time²	0.00023	0.00031	0.74956	.45510		

Note. Robust linear mixed effects regression models for aggregation parameters. Sign. quadratic change in EL max, No sign. change in SS ½, SS ½/EI max

Robust Models: Cubic

		b	**SE**	***t***	***p***	***R²* cond. [marginal]**	***RMSE***
**EI max**	(Intercept)	0.489	0.004	127.573	0.00000	0.352 [0.040]	0.019
	time	-0.004	0.002	-2.171	0.032		
	time²	0.000	0.000	1.551	0.124		
	time³	0.000	0.000	-1.329	0.186		
**SS ½ individual**	(Intercept)	1.12857	0.02917	38.68386	0.00000	0.373[0.019]	0.150
	time	-0.02784	0.01530	-1.81964	0.07155		
	time²	0.00335	0.00204	1.64409	0.10303		
	time³	-0.00008	0.00005	-1.53691	0.12720		
**SS ½/EI max**	(Intercept)	2.31328	0.04929	46.93515	0.00000	0.409 [0.013]	0.254
	time	-0.03859	0.02502	-1.54226	0.12594		
	time²	0.00501	0.00334	1.50154	0.13613		
	time³	-0.00012	0.00009	-1.44314	0.15187		

Aggregation:

Isc max (au)

Difference between CON and aCOV T0, aCOV T2, aCOV T5, aCOV T10, aCOV T30. Assumptions of linear regression visually checked => can be assumed

$F(5, 40.6) = 1.28$, $p = .292$, ES = 0.34 [0.16; 0.53]

	b	*SE*	95 % - CI	95 %- CI	*t*	df $_{error}$	*p*
**(Intercept)**	34.25	0.83	32.61	35.89	41.22	183	<.001
**CON_vs_aCOV T0**	0.39	1.22	-2.02	2.8	0.32	183	.749
**CON_vs_aCOV T2**	2.38	1.24	-0.06	4.83	1.92	183	.056
**CON_vs_aCOV T5**	2.73	1.27	0.22	5.24	2.15	183	.033
**CON_vs_aCOV T10**	0.14	1.5	-2.82	3.1	0.09	183	.925
**CON_vs_aCOV T30**	1.82	1.84	-1.81	5.45	0.99	183	.324

AI (%)

Difference between CON and aCOV T0, aCOV T2, aCOV T5, aCOV T10, aCOV T30. Assumptions of linear regression visually checked: not given, robust models.

$F(5, 36.76) = 3.95$, $p<.001$, ES =0.4 [0.22; 0.59]

	b	*SE*	95 % - CI	95 %- CI	*t*	df $_{error}$	*p*
**(Intercept)**	59.11	1.43	56.3	61.93	41.44	183	<.001
**CON_vs_aCOV T0**	6.72	2.07	2.64	10.8	3.25	183	.001
**CON_vs_aCOV T2**	3.24	2.12	-0.95	7.42	1.53	183	.129
**CON_vs_aCOV T5**	1.24	2.2	-3.11	5.58	0.56	183	.576

**CON_vs_aCOV T10**	0.93	2.67	-4.34	6.2	0.35	183	.728
**CON_vs_aCOV T30**	0.35	3.16	-5.87	6.58	0.11	183	.911

Multilevel models:

Prediction of AI by ZP. Analyzed using multilevel model.

Assumptions of linear regression visually checked. No NV of residuals available.

Robust linear mixed effects regression (Koller, M. robustlmm: An R Package for Robust Estimation of Linear Mixed-Effects Models. J. Stat. Softw. 2016, 75, 1–24. https://doi.org/10.18637/jss.v075.i06.). P-values calculated from t-statistics of the robust estimate and df of the equivalent "normal" model.

Shr at dIsc min (1/s)

Difference between CON and aCOV T0, aCOV T2, aCOV T5, aCOV T10, aCOV T30. Assumptions of linear regression visually checked cannot be assumed; use robust model.

$F(5, 34.12) = 17.64$, $p <.001$, ES = 0.48 [0.32; 0.75]

	b	***SE***	**95 % - CI**	**95 %- CI**	***t***	**df** $_{error}$	***p***
**(Intercept)**	119.67	19.27	81.65	157.68	6.21	183	<.001
**CON_vs_aCOV T0**	139.41	29.52	81.17	197.64	4.72	183	<.001
**CON_vs_aCOV T2**	131.85	27.78	77.04	186.65	4.75	183	<.001
**CON_vs_aCOV T5**	70.5	31.04	9.25	131.75	2.27	183	.024
**CON_vs_aCOV T10**	98.1	34.56	29.92	166.28	2.84	183	.005
**CON_vs_aCOV T30**	53.67	41.95	-29.11	136.44	1.28	183	.202

T ½ [$^1/_s$]

Difference between CON and aCOV T0, aCOV T2, aCOV T5, aCOV T10, aCOV T30. Assumptions of linear regression visually verified not given: interpret robust model. F(5, 36.8) = 4.79, p <.010, ES = 0.42 [0.22; 0.66]

	b	*SE*	95 % - CI	95 %- CI	*t*	df $_{error}$	*p*
**(Intercept)**	2.77	0.2	2.37	3.16	13.64	183	<.001
**CON_vs_aCOV T0**	-0.85	0.29	-1.43	-0.27	-2.89	183	.004
**CON_vs_aCOV T2**	-0.32	0.3	-0.91	0.26	-1.09	183	.277
**CON_vs_aCOV T5**	0.23	0.31	-0.38	0.83	0.75	183	.456
**CON_vs_aCOV T10**	0.2	0.36	-0.52	0.91	0.54	183	.590
**CON_vs_aCOV T30**	-0.04	0.44	-0.9	0.83	-0.08	183	.935

Time development: Aggregation

Multilevel models:

Prediction of AV by ZP and ZP². Analyzed by multilevel model.

Assumptions of linear regression visually checked. No NV of the residuals available. Robust linear mixed effects regression (Koller, M. robustlmm: An R Package for Robust Estimation of Linear Mixed-Effects Models. J. Stat. Softw. 2016, 75, 1–24. https://doi.org/10.18637/jss.v075.i06.). P-values calculated from t-statistics of the robust estimate and df of the equivalent "normal" model.

Quadratic Models

		b	SE	*t*	*p*	*R²* cond. [marginal]	*RMSE*
**Isc max**	(Intercept)	35.236	0.840	41.928	< .001	.543 [.009]	4.238
	time	0.217	0.151	1.435	.154		
	time²	-0.008	0.005	-1.527	.130		
**AI [%]**	(Intercept)	65.040	1.466	44.373	< .001	.615 [.057]	6.915
	time	-0.968	0.247	-3.917	< .001		
	time²	0.027	0.008	3.235	< .010		
**Shr at dIsc min**	(Intercept)	278.321	19.077	14.589	< .001	.535 [.054]	132.494
	time	-11.015	3.583	-3.074	< .010		
	time²	0.271	0.118	2.290	< .050		
**T ½ (¹/ₛ)**	(Intercept)	2.003	0.170	11.748	< .001	.590 [.065]	0.993
	time	0.122	0.030	4.048	< .001		
	time²	-0.003	0.001	-3.338	< .001		

Note. Robust linear mixed effects regression models for aggregation parameters

Significant quadratic time effect for AI, Shr at Disc and t ½ could be found, indicating a quadratic change of these parameters over time.

For Isc max there was no sign. effect of time: on average no change of Isc max over time could be found

Cubic Models:

		b	SE	*t*	*p*	*R²* cond. [marginal]	*RMSE*
**Isc max**	(Intercept)	34.5	0.88	39.26	<.001	0.556[0.027]	4.183
	time	1.04	0.4	2.63	.010		

	time^2	-0.12	0.05	-2.36	.020		
	time^3	0.00	0.00	2.22	.029		
**AI [%]**	(Intercept)	66.13	1.54	42.97	<.001	0.636 [0.070]	6.85
	time	-2.36	0.65	-3.66	<.001		
	time^2	0.23	0.09	2.66	.009		
	time^3	-0.01	0.00	-2.38	.020		
**Shr at dIsc min**	(Intercept)	287.63	20.29	14.18	<.001	0.539 [0.060]	131.774
	time	-21.95	9.37	-2.34	.021		
	time^2	1.84	1.26	1.46	.146		
	time^3	-0.04	0.03	-1.25	.215		
**T ½ ($^1/_s$)**	(Intercept)	1.89	0.18	10.45	<.001	0.609 [0.074]	0.967
	time	0.27	0.08	3.48	.001		
	time^2	-0.03	0.01	-2.41	.018		
	time^3	0.00	0.00	2.11	.038		

Table S3: Descriptive Statistics and statistical results of erythrocyte staining intensities

	aCov (N= 9)				Con (N =28[a] bzw. 29[b])					
	M (SD)	25% - Quantil	75%- Quantil	Median	M (SD)	25% - Quantil	75%- Quantil	Median	W	p
Immuno 1177[a]	6.14 (2.47)	2.85	8.25	6.64	6.72 (2.91)	4.46	8.22	6.84	139	.658
Nitro[b]	14.48 (7.47)	8.92	18.95	12.89	4.75 (2.32)	2.95	6.08	4.43	14	5.914e-06

Comparison between aCov and healthy controls (Con) was possible for t0, only.

Unpaired Wilcoxon tests were used to determine the difference between aCov at t0 and Con.
